# Aquaporin-4 Functionality and Virchow-Robin Space Water Dynamics: Physiological Model for Neurovascular Coupling and Glymphatic Flow

**DOI:** 10.3390/ijms18081798

**Published:** 2017-08-18

**Authors:** Tsutomu Nakada, Ingrid L. Kwee, Hironaka Igarashi, Yuji Suzuki

**Affiliations:** 1 Center for Integrated Human Brain Science, Brain Research Institute, University of Niigata, Niigata 951-8585, Japan; ilkwee@ucdavis.edu (I.L.K.); higara@bri.niigata-u.ac.jp (H.I.); yuji-s@bri.niigata-u.ac.jp (Y.S.); 2 Department of Neurology, University of California, Davis, VANCHCS, Martinez, CA 94553, USA

**Keywords:** Hagen-Poiseuille equation, starling resister, rCBF, interstitial flow, glia limitans externa

## Abstract

The unique properties of brain capillary endothelium, critical in maintaining the blood-brain barrier (BBB) and restricting water permeability across the BBB, have important consequences on fluid hydrodynamics inside the BBB hereto inadequately recognized. Recent studies indicate that the mechanisms underlying brain water dynamics are distinct from systemic tissue water dynamics. Hydrostatic pressure created by the systolic force of the heart, essential for interstitial circulation and lymphatic flow in systemic circulation, is effectively impeded from propagating into the interstitial fluid inside the BBB by the tightly sealed endothelium of brain capillaries. Instead, fluid dynamics inside the BBB is realized by aquaporin-4 (AQP-4), the water channel that connects astrocyte cytoplasm and extracellular (interstitial) fluid. Brain interstitial fluid dynamics, and therefore AQP-4, are now recognized as essential for two unique functions, namely, neurovascular coupling and glymphatic flow, the brain equivalent of systemic lymphatics.

## 1. Introduction: Blood-Brain Barrier

The blood-brain barrier (BBB) is the result of unique properties of brain capillary endothelium, namely the presence of tight junctions and active suppression of aquaporin-1 (AQP-1) expression, the water channel abundantly expressed in common capillaries and choroid plexus epithelium [1,2,3,4]. The BBB effectively isolates the brain from the systemic environment through its selectivity. Brain capillary endothelium has no fenestrations, and has highly restricted permeability to ions and water as evidenced by a significantly high electrical resistance [5,6]. There is virtually no non-specific transcellular and paracellular diffusion of hydrophilic compounds across the BBB. Instead, brain capillary endothelium has specifically localized receptors and transporters responsible for the active transport of nutrients to the brain interstitium.

**Figure 1:**
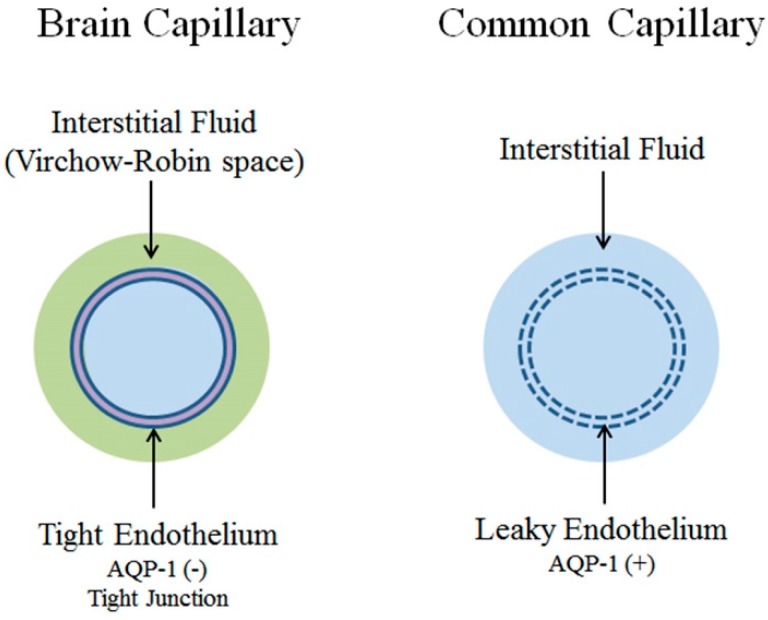
Capillaries. Common capillaries have a leaky endothelium due to the presence of fenestrations. The water channel aquaporin-1 (AQP-1) is also abundantly expressed. Accordingly, water dynamics between intracapillary and interstitial fluid space is directly connected (right). In contrast, brain capillaries lack fenestrations and have tight junctions. Expression of AQP-1 is actively suppressed. As a result, water dynamics in the intracapillary space and interstitial fluid space are effectively isolated from each other and must be analyzed independently. Modified from Reference [9].

Tight junctions of the brain endothelial cells are structurally similar to epithelial tight junctions. The major transmembrane proteins in tight junctions include occludins and claudins [5]. While some accessory proteins specific to brain endothelium, such as cingulin, AF-6 and 7H6, have been identified, other epithelial tight junction proteins have thus far not been detected. Epithelial tight junctions, including choroid plexus epithelial cells, mostly contain claudin-1, -2 and -11. By contrast, brain endothelial tight junctions primarily express claudin-3 and -5. Accumulating evidence indicates that claudin-3 and -5, together with occludin, is responsible for controlling BBB paracellular permeability [5,6]. Thus far, only claudin-2, which is found on so-called leaky epithelium but not brain endothelium, has been shown to be a water channel [7]. Considering that expression of AQP-1, abundant in common capillaries and choroid plexus epithelium, is actively suppressed in brain capillary endothelium [2,3], it is consonant that claudin-2 is also absent in the tight junctions of brain capillary endothelium (Figure 1).

Studies to date have not identified any specific passage for water to the BBB. It appears that water entry into the inside of the BBB is non-specific, presumably slow movement through the lipid membrane [8]. It follows that brain fluid dynamics would be distinct from systemic fluid dynamics. We present here a brief review of the recent findings on the physiological and molecular mechanisms of brain fluid dynamics and its impact on two essential processes, namely, neurovascular coupling and glymphatic flow.

## 2. Blood Flow Dynamics

### 2.1. Basics

Classic blood flow dynamics can be given by the Hagen-Poiseuille equation where volumetric blood flow, Φ, is given as:

$$\Phi =\frac{\pi }{8\eta }\frac{\Delta P}{L}R^{4}$$

where Δ*P* is pressure loss (differences in inflow and outflow pressure), *L* is the length of the vessel tube, *η* is blood viscosity, and *R* is the radius of the vessel [10].

Under physiological conditions, *L* and *η* can be treated as constant, and regional blood flow, Φ_*r*_, within the unit area can be given as:

$$\Phi_{r}\sim \Delta P\cdot R^{4}$$

In the systemic circulation, the autonomic nervous system is responsible for Φ*r* by controlling both ∆*P* and *R* through neural and/or chemical control of contractile structures of cardiac and circulatory systems. In contrast, capillaries lack contractile structures and, therefore, capillary blood flow cannot be directly controlled by the autonomic nervous system. Although contractile function of pericytes and its neurotransmitter control has been proposed [11], considering its common, wide spread functionality related to angiogenesis and stem cell like behavior [12], it is difficult to accept that pericytes are the primary structural component for flow regulation. It is, therefore, highly plausible to consider that capillary flow dynamics are a passive phenomenon which are significantly affected by the architectural properties of the capillaries per se.

### 2.2. Common Capillaries and Tissue Perfusion

Common capillaries in the systemic circulation have a leaky endothelium. Water flow between intra-capillary and interstitial fluid is relatively free and follows the forces defined by the Starling equation:

$$J_{v}=L_{p}S\left(\left[P_{c}-P_{i}\right]-\sigma \left[\pi_{p}-\pi_{i}\right]\right)$$

where *J_v_* is the trans endothelial fluid filtration volume per second, *P_c_* is the capillary hydrostatic pressure, *P_i_* is the interstitial hydrostatic pressure, *π_p_* is the plasma protein oncotic pressure, *π_i_* is the interstitial oncotic pressure, *L_p_* is the hydraulic conductivity of the membrane, *S* is the surface area for filtration, and *σ* is the Staverman’s reflection coefficient, respectively [13].

The term $\left[P_{c}-P_{i}\right]-\sigma \left[\pi_{p}-\pi_{i}\right]$ in the Starling equation represents the net driving force for the trans endothelial fluid filtration volume per second, *J_v_*. Under physiological conditions, $\sigma \left[\pi_{p}-\pi_{i}\right]$ and *P_i_* will be virtually constant and, therefore, regional flow per second, Φ_*r*_ per second, and hence averaged ∆*P* per second, will directly translate into *J_v_*, defining tissue perfusion. Simply stated, for systemic, leaky capillaries, *R* remains constant, *K_r_*.

$$\Phi_{r}\sim \Delta P\cdot R^{4}$$

$$R=K_{r}$$

Accordingly, Φ_*r*_ is a function of ∆*P*.

$$\Phi_{r}\sim \Delta P$$

Therefore, as far as tissues with common capillaries are concerned, the hydrostatic pressure field generated by the heart is by far the most important, if not sole, factor governing tissue perfusion. The systolic force of the heart effectively extrudes water out of the capillaries without much resistance into the interstitial fluid system as well as helps create significant interstitial fluid motion necessary for interstitial fluid circulation (see below).

In clear contrast, brain capillaries have significantly different water permeability characteristics due to the presence of the BBB. Therefore, the principles of regional blood flow and interstitial fluid circulation described for common capillaries are not applicable to regional cerebral blood flow (rCBF).

### 2.3. Cerebral Autoregulation

Cerebral autoregulation signifies an intrinsic ability of cerebral vasculature to maintain cerebral blood flow at a relatively constant rate of ca. 50 mL per 100 g brain tissue per minute in the face of blood pressure changes [14,15,16,17,18,19]. Autoregulation generally functions between mean blood pressures of 60 and 150 mm Hg. It is preserved in animals that have undergone parasympathetic and/or sympathetic denervation [14], and the system is independent from extrinsic neural control. Instead, intrinsic neural nitric oxide (NO) control [19] and release of vasoactive substrates by the brain are believed to play essential roles in maintaining constant cerebral perfusion [16,17]. Perfusion is held constant by means of cerebral vasculature smooth muscle constriction and dilation in response to elevated and decreased systemic pressure, respectively [14,15,16,17,18,19].

In the context of the principles of the blood flow dynamics described above, cerebral autoregulation translates to rigorous maintenance of a constant ∆*P* (differences in inflow and outflow pressure), *K_p_*.

$$\Phi_{r}\sim \Delta P\cdot R^{4}$$

$$\Delta P=K_{p}$$

If brain capillaries have structural properties similar to common capillaries, rCBF would be essentially constant.

$$\Phi_{r}\sim \Delta P\sim K_{p}$$

This is indeed the primary functionality of cerebral autoregulation, which rigorously maintains a rather constant perfusion of the brain in the presence of significant fluctuation in systemic circulation associated with various physiological activities [14,15,16,17,18,19].

### 2.4. Neurovascular Coupling

Increased rCBF associated with brain activation is a well-recognized phenomenon that is known as neurovascular coupling. It is relatively small increase in rCBF compared to steady rCBF, which is rigorously regulated by autoregulation [20]. Nevertheless, the rCBF increase associated with neurovascular coupling seemingly contradicts the purpose of cerebral autoregulation and, therefore, neurovascular coupling should have a role essential to maintain brain functionality and closely related molecular phenomena associated with neural activities.

It was once thought that neurovascular coupling serves to ensure adequate neuronal nutrient supply. This intuitively appealing notion failed to account for the large quantitative discrepancy between supply and demand. The amount of essential nutrients delivered by the increased rCBF, such as oxygen and glucose, exceeds actual consumption by more than six times. Such a large discrepancy is virtually unknown in any other biological system, indicating that a factor other than nutrient supply underlies the observed disproportionate increase in rCBF [20]. Subsequent studies indicate that increased rCBF associated with brain activation serves as a heat removal mechanism. Information processing by brain generates considerable heat and water flow is the primary means of heat removal. It is therefore evident that the apparent surfeit in rCBF increase serves as a quick removal system of the additional heat generated by neural activities [21,22].

Neurovascular coupling is a micro, rather than macro environmental event occurring within an area limited to 250 µm around the site of neural activity [23]. Therefore, the regulatory mechanism for neurovascular coupling should be within the capillaries, independent from cerebral autoregulation per se. Brain capillaries have a very tight endothelium which severely restricts water permeability. Therefore, hydrostatic pressure differences between intra-capillary fluid and interstitial fluid affects the capillary’s structure as in the case of a Starling resistor [24]. This can provide the environment where small rCBF increase associated with neural activities, Φ_*nvc*_, under rigorous control of rCBF by autoregulation is a function of small changes in capillary diameter ∂*R*.

$$\Phi_{nvc}\sim \partial R^{4}$$

### 2.5. Flow and Pressure in a Starling Resistor

**Figure 2:**
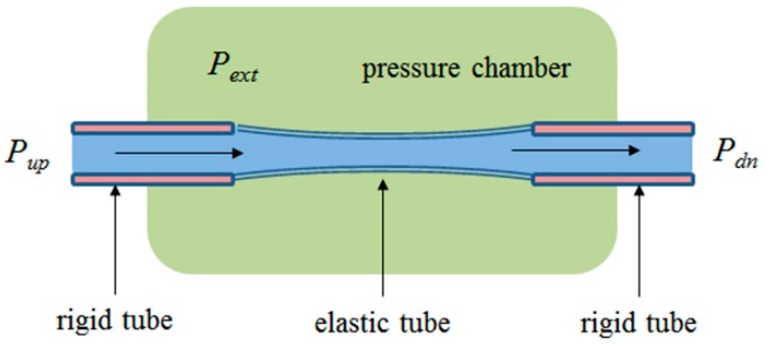
The experimental set-up known as a Starling resistor. An elastic tube clamped between rigid tubes is surrounded by an outer pressure chamber. Flow is driven through the tube from left to right by an applied pressure differences, *P_up_*-*P_dn_*. The pressure of the chamber, *P_ext_*, can be adjusted to control the average diameter of the elastic tube. *P_up_*: upstream pressure; *P_dn_*: downstream pressure; *P_ext_*: external chamber pressure.

The experimental device consisted of an elastic tube clamped between two rigid pipes, surrounded by an outer pressure chamber, known as a Starling resistor, and was used in an isolated-heart preparation by Ernest Henry Starling. Studies using the device eventually led to the Frank-Starling law of the heart [13,24] (Figure 2). The static pressure of the outer pressure chamber controls the degree of collapse of the tube, providing a variable resistor to simulate total peripheral resistance. As a rule, the device initially shows linear behavior, namely, linear changes in tube diameter that corresponds to the relationship between the pressure of the outer chamber and hydrodynamic pressure in the tube. It eventually shows non-linear behavior, leading to two non-linear phenomena known as the waterfall effect and self-excited oscillations, the biological equivalents of which represent expiratory flow limitation in chronic obstructive pulmonary disease (COPD) and snoring, respectively [25].

Brain capillaries, owing to tight endothelium, have virtually identical physiological conditions as a Starling resistor. The brain capillary is a biological pliable tube clamped between two rigid tubes, namely, arteriole (artery) and venule (vein) as depicted in Figure 2. Cerebral autoregulation rigorously maintains hydrodynamic pressure corresponding to *P_up_*-*P_dn_*. Similar to the elastic tube in a Sterling resistor, the diameter of which is controlled by the pressure of the outer pressure chamber, *P_ext_*_,_ in Figure 2. The diameter of a given brain capillary, *R*_cap_, is a function of the static pressure of the surrounding interstitial fluid in the peri-capillary space (Virchow-Robin space), *P_VRS_*.

Rcap~1PVRS

## 3. AQP-4 and Neurovascular Coupling

### 3.1. Virchow-Robin Space and Interstitial Flow

Fluid-filled canals surrounding perforating arteries, capillaries, and veins in the brain parenchyma were recognized early in modern medicine and are referred to as the Virchow-Robin space (VRS), based on the first two scientists who described the structures in detail, Rudolph Virchow and Charles Philippe Robin [26,27]. It was soon recognized that interstitial fluid in VRS was dynamic, and this fluid movement was termed interstitial flow. Interstitial fluid in the peri-capillary space moves along the perforating artery and vein and eventually drains into the cerebrospinal fluid (CSF) system.

**Figure 3:**
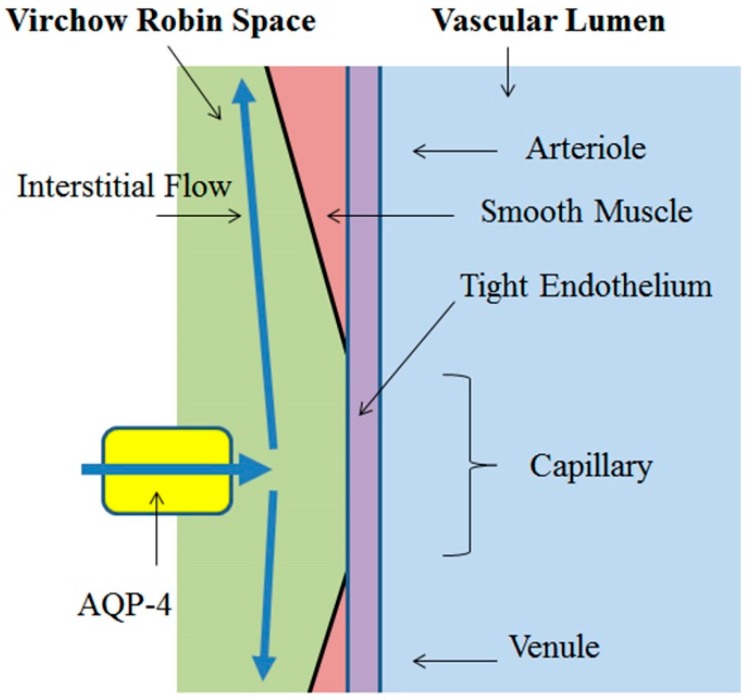
Peri-capillary VRS water dynamics. Brain capillary endothelial tight junctions and active suppression of AQP-1 expression highly restricts water movement across the BBB. By contrast, significant water flow (blue arrow) is present inside the BBB within VRS (interstitial flow), mediated by active water inflow through AQP-4. Modified from Reference [9].

Unlike common capillaries, water flow from brain capillary to VRS is significantly restricted and not sufficient for generating interstitial flow. Recent studies disclosed that water dynamics in peri-capillary VRS and, hence, interstitial flow, is maintained by aquaporin-4 (AQP-4), a water channel abundantly expressed at the endfeet of peri-capillary astrocytes. AQP-4 connects the intracellular space of astrocytes and extracellular (interstitial) space at capillary VRS, thereby regulating water efflux essential for peri-capillary VRS water dynamics [28,29,30]. As discussed above, peri-capillary VRS hydrostatic pressure, *P_VRS_*, is the determinant of brain capillary diameter, *R*_cap_. It follows that AQP-4 activity is also the main factor governing brain capillary diameter (Figure 3).

Rcap~1PVRS~1AQP4 Activities~AQP4 Supression

Since rCBF increases by a factor of four of the capillary diameter, $\Phi_{r}\sim R^{4}$, rCBF increases significantly when there is a minor reduction in AQP-4 activities, which consequently results in expansion of capillary diameter. Indeed, the AQP-4 inhibitor, TGN-020, was found to increase rCBF significantly [31].

### 3.2. Neural Activities and AQP-4 Suppression

The role of AQP4 in neural signal transduction was first proposed following the observation that deletion of α-syntrophin in mice is associated with prolonged potassium (K^+^) clearance [32]. In the hippocampus, the rapid water fluxes driven by AQP-4 are found to be required for maintaining K^+^ homeostasis during electrical activity [24]. Inwardly rectifying potassium channels, Kir4.1, responsible for the rapid removal of K^+^ from extracellular (interstitial) fluid essential for maintaining neural excitability, are co-localized with AQP-4 on astrocyte endfeet facing capillary endothelium at the VRS [33]. This K^+^ spatial buffer mechanism is responsible for the rapid redistribution of K^+^ by a current flow termed potassium siphoning.

K^+^ entry generates a local depolarization, which propagates electrotonically through individual glial cells and through the glial cell syncytium [34,35]. The overall efficiency of the spatial buffer process is thought be dependent, in part, on the electrical space constant of the glial cell syncytium. However, it is known that potassium siphoning within a single glial cell is sufficient. K^+^ influx occurring in one region of the glial cell is balanced by efflux at another region of the astrocyte, typically, at the endfeet. While K^+^ influx occurs primarily through Kir, K^+^ efflux occurs through the gap junctions [34,35,36,37]. Kir distribution corresponds to the highly polarized localization of AQP-4 [32,33,34].

Kir4.1 and AQP-4 are considered to belong to the dystrophin-glycoprotein complex (DGC) family [34]. The HCO_3_^−^ independent proton pump, vacuolar ATPase (V-ATP), expressed abundantly in astrocytes, is also considered part of the DGC family [38]. The functional coupling of Kir, AQP-4 and V-ATP with respect to neural activation has become evident [9]. As suggested by Hibino and Kurachi [39], this co-localization may be the result of a clustering of proteins in specialized lipid raft domains which bring together functionally coupled molecules to a common site on the membrane. The implications of disruption in this coupled functionality, including AQP-4, with respect to neural functionality are only beginning to be understood.

**Figure 4:**
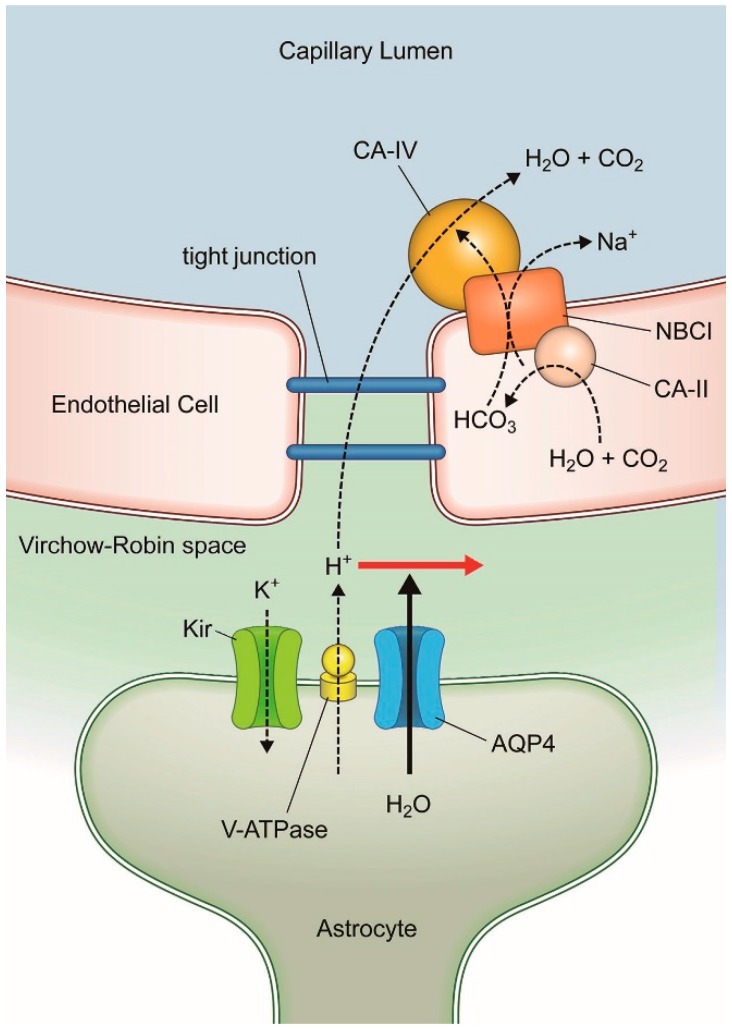
Molecular processes within the Virchow–Robin space associated with neural activation. Neural activation induces potassium efflux into the interstitial space which is quickly scavenged by inwardly rectifying potassium channel (Kir). This potassium spatial buffering promotes release of a counterpart cation, H^+^, by vacuolar ATPase, resulting in proton excess in the interstitial fluid (extracellular acidosis). AQP-4 is inhibited by higher proton density (red arrow). Excess protons move into the intracapillary space without significant resistance at the tight junctions because of the Grotthuss proton tunneling mechanism. Carbonic anhydrase type IV (CA-IV) anchored to the luminal surface of capillaries and NBC1 sodium bicarbonate co-transporter that interacts directly with CA-IV effectively scavenge these excess protons. Modified from Reference [9].

AQP-4 suppression associated with neural activities can be seen as part of a cascade initiated by rapid K^+^ flux into the extracellular space by production of action potentials. Rapid spatial buffering by Kir promotes release of its counterpart cation, H^+^, by V-ATP, resulting in extracellular acidification. The increase in extracellular H^+^ inhibits AQP-4 activity at VRS [40] (Figure 4).

### 3.3. AQP-4 Suppressionand Neurovascular Coupling

**Figure 5:**
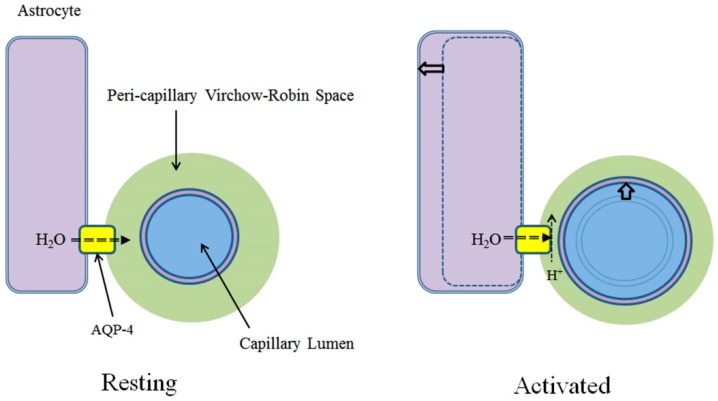
At the VRS, neural activation produces extracellular acidification accompanied by increase in rCBF and astrocyte swelling. Proton inhibition of AQP-4 results in a reduction of water flow from astrocytes into the peri-capillary Virchow-Robin space, reduction of peri-capillary fluid pressure, capillary lumen expansion, increase rCBF and astrocyte swelling. Modified from Reference [9].

AQP-4 suppression initiated by neural activities which triggers rapid K^+^ flux into the extracellular space described above is the likely underlying molecular mechanism of neurovascular coupling. Indeed, in humans, the area of extracellular acidification associated with regional neural activity is confirmed to be identical to the area of rCBF increase [41]. A decline in interstitial fluid hydrostatic pressure due to suppression of AQP-4 results in caliber expansion of the tightly sealed brain capillaries and, hence, increased rCBF. This phenomenon also explains a curious co-phenomenon, namely, astrocyte swelling associated with neural activities. AQP-4 suppression by extracellular proton excess leads to relative intracellular water excess, in turn producing astrocyte swelling. This phenomenon has been shown to be absent in AQP-4 knock out mice [42] (Figure 5).

## 4. AQP-4 and Interstitial Fluid Circulation

### 4.1. Interstitial Flow as Brain Lymphatic Equivalent–Glymphatic Flow

Interstitial flow has been postulated to play a role similar to systemic lymphatics for the brain, which lacks a conventional lymphatic system [43,44,45,46]. The concept has recently been revived in relation to CSF dynamics and β-amyloid clearance [43,44,47,48]. It is now clear that the classic view of CSF circulation where the choroid plexus is solely responsible for CSF production is invalid [49]. Although choroid plexus also produces CSF, interstitial fluid enters the CSF system and contributes to CSF volume significantly [29,49]. This brain lymphatic equivalent system is now widely referred to as glymphatics [48,50], denoting glial lymphatics.

As the lymphatic equivalent of the brain, the glymphatic system is anticipated to have a functional configuration similar to that of the systemic lymphatic system [51]. In the systemic circulation where common, leaky capillaries exist, hydrostatic pressure generated by the systolic force of the heart effectively extrudes water out of the capillaries without much resistance into the interstitial fluid space, and helps create interstitial fluid motion necessary for interstitial circulation [13,15]. Lymphatic capillaries have primary valves which prevent backflow effecting one way flow. The hydrostatic pressure created by the systolic force of the heart is usually sufficient to return interstitial fluid to the systemic circulation although an occasional build-up of interstitial fluid can occur causing edema, especially in areas vulnerable to gravitational force [13,51].

**Figure 6:**
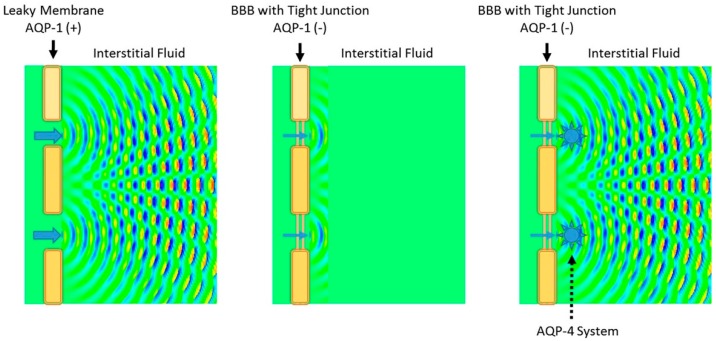
Schematic presentation of wave propagation simulation for conceptualizing water dynamics inside the BBB. From a hydrodynamic standpoint, the tight endothelium of brain capillaries forming the BBB is seriously flawed. While hydrostatic pressure generated by the systolic force of the heart (blue arrow) provides the necessary kinetics in the case of fenestrated capillaries (**left**), sealed capillaries impede propagation of such a force (**middle**). The addition of AQP-4 driven water flow reconstitutes the necessary environment (**right**). Diagrams are created using wave propagation/interference simulation for two windows.

In contrast, the endothelium of brain capillaries is effectively sealed by tight junctions, the primary structure responsible for the BBB [1,2,3,4]. Active suppression of AQP-1 expression in brain capillaries further ensures restriction of water movement across the BBB [5]. From a hydrostatic dynamics standpoint, the BBB also impedes propagation of the systolic force of the heart across capillary endothelium. Accordingly, the combined restricted water mobility from capillaries to the interstitial space of the brain and blocked hydrostatic force are insufficient to produce interstitial fluid movement within the BBB. Therefore, to achieve interstitial fluid dynamics in the brain similar to that of systemic lymphatics, additional mechanisms for increasing water volume/contents and hydrostatic pressure within the peri-capillary interstitium are essential to ensure glymphatic flow. Water flux through AQP-4 into peri-capillary VRS provides the requisite water flow within the peri-capillary interstitium (Figure 6).

### 4.2. Glymphatic Flow and β-Amyloid Clearance

The concept that glymphatic flow is the systemic lymphatic equivalent, in relation to CSF dynamics and β-amyloid clearance, has recently become a major focus of medical investigation [30,43,47,50,52]. It is now clear that glymphatic flow within VRS is responsible for clearance of β-amyloid, and disturbance in glymphatic flow likely plays an important role in the pathogenesis of Alzheimer’s disease (AD) [30,50,52]. Subsequently, it has been confirmed that patients with AD indeed have impaired glymphatic flow [53].

Glymphatic flow is dependent on AQP-4 function for β-amyloid clearance. In animal studies, conditions which can produce potential decline in AQP-4 function all result in β-amyloid accumulation [44,45,46,47,48,49,50,51,52]. In humans, anti-AQP-4 antibody positive autoimmune neuromyelitis optica (NMO) does not affect AQP-4 functionality per se. Consonant with this, there is no β-amyloid accumulation observed in anti-AQP-4 antibody positive autoimmune NMO [54]. In contrast, Duchenne muscular dystrophy (DMD) is accompanied by AQP-4 dysfunction and also associated with excess β-amyloid accumulation [55]. DMD patients studied by single photon emission computed tomography (SPECT) demonstrated GABA-A abnormalities [56], a finding consonant with the known β-amyloid impairment of synaptic inhibition via GABA-A receptor endocytosis [57].

### 4.3. AQP-4 as Interstitial Fluid Circulator

**Figure 7:**
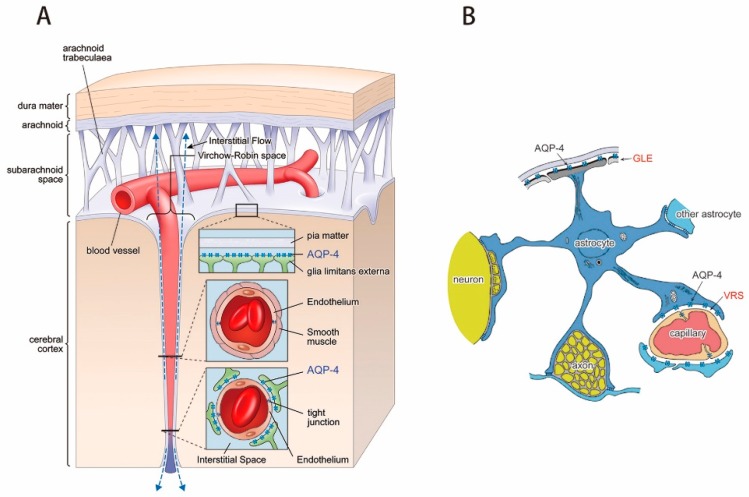
Astrocyte and polarized localization of AQP-4. (**A**): Expression of AQP-4 in the brain is highly polarized to endfeet of astrocytes at two specific locations, the glia limitans externa (GLE) at the cortical surface and peri-capillary Virchow-Robin space (VRS). VRS constitutes fluid-filled canals surrounding perforating arteries, capillaries, and veins in brain parenchyma. While pia mater ends near the brain surface, VRS continues into the brain parenchyma accompanying a perforating artery; (**B**): Astrocyte endfeet attach to many structures. However, AQP-4 is found only at the GLE and VRS.

AQP-4 in the brain is highly localized to astrocyte endfeet at the glia limitans externa (GLE) and peri-capillary VRS (Figure 7) [58,59,60]. This polarized location of AQP-4 expression is frequently subject of erroneous speculation that AQP-4 facilitates the flow of water into and out of the brain [60]. As discussed above, the existence of the BBB precludes this possibility [1,2,3,4]. Indeed, the absence of AQP-4 does not alter water entry into the BBB from the systemic circulation, although it does reduce water entry into the CSF system to a level similar to brain parenchyma [29]. Polarized localization of AQP-4, therefore, has another functionality for water dynamics within the BBB.

**Figure 8:**
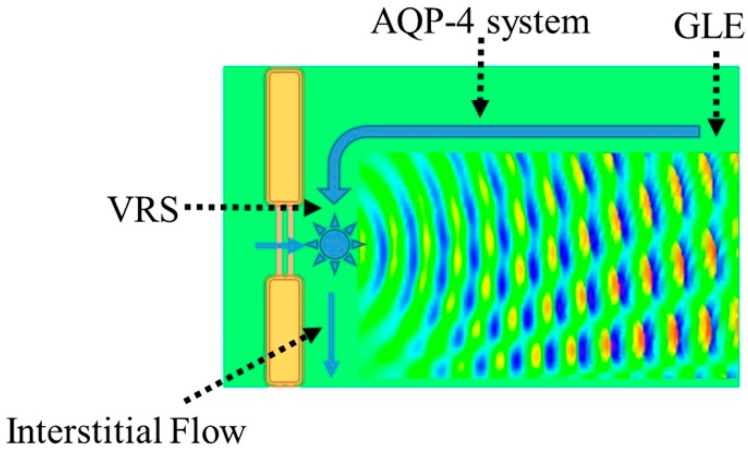
AQP-4 circulator model. The AQP-4 system provides additional water flow into peri-capillary VRS (thick blue arrow). Water enters astrocytes through AQP-4 at the GLE. This internal circulation further promotes appropriate interstitial fluid dynamics including flow through VRS (interstitial flow), otherwise known as glymphatic flow.

AQP-4 at peri-capillary VRS connects astrocyte intracellular space and extracellular (interstitial) space. Water efflux from astrocytes into the interstitial space must be balanced by an equivalent amount of water influx in order to maintain water equilibrium. Since astrocyte AQP-4 is also localized to endfeet at the GLE, it is credible that water movement from the interstitial space into intracellular space of astrocytes occurs at the GLE. In this schema, the AQP-4 system functions as circulator of interstitial fluid within the BBB, and is responsible for producing interstitial fluid movement essential for glymphatic flow (Figure 8).

The AQP-4 system appears to serve to facilitate water dynamics within the BBB. In addition to providing additional water into the peri-capillary space, this system also creates a water density gradient within the interstitial space further promoting movement of interstitial fluid to the surface of the brain. Indeed, 7T MRI microscopic analysis of the relaxation properties of water molecules in humans demonstrated that the cortical area around GLE has a lower water density, effectively creating an electrostatic environment [61].

## 5. Conclusions

The tight endothelium of brain capillaries plays a critical role in maintaining the BBB, a unique functional property of the brain. Water permeability through the BBB is highly restricted through suppression of expression of AQP-1 and the presence of tight junctions. As a result, brain water dynamics is effectively isolated from the systemic circulation, and AQP-4, a water channel abundantly expressed at the endfeet of astrocytes, plays a critical role in the water dynamics inside the BBB. Two unique functional features characterize AQP-4 facilitated brain water dynamics, namely neurovascular coupling and glymphatic flow. The former is initiated by neural activities that trigger rapid K^+^ flux into the extracellular space that eventually results in extracellular acidosis and AQP-4 suppression, which in turn reduces interstitial pressure, increases capillary diameter and leads to an increase in rCBF. The latter occurs in spite of the tight endothelium of brain capillaries which effectively impedes propagation of the hydrostatic pressure created by the systolic force of the heart to the interstitial fluid inside the BBB. Functionality of AQP-4 is necessary for water influx into peri-capillary VRS and the generation of interstitial fluid dynamics essential for glymphatic flow, the lymphatic equivalent in the brain. Considering that β-amyloid clearance is dependent on glymphatic flow, proper AQP-4 functionality is likely a key factor in the prevention and/or treatment of AD.
